# Automated Stratum Interface Detection Using the Optimized Drilling Specific Energy through Self-Adaptive Logistic Function

**DOI:** 10.3390/s23208594

**Published:** 2023-10-20

**Authors:** Kechen Liu, Jingyi Cheng, Xin Sun, Xiang Li, Zhijun Wan, Keke Xing, Jianzhuang Liu

**Affiliations:** 1 School of Mines, China University of Mining and Technology, Xuzhou 221116, China; 01170230@cumt.edu.cn (K.L.); tb21020042b0@cumt.edu.cn (X.S.); zhjwan@cumt.edu.cn (Z.W.); tb20020032b4@cumt.edu.cn (K.X.); 2 Hebei Provincial Key Laboratory of Mining Development and Safety Engineering, North China University of Science and Technology, Tangshan 063009, China; cumtljz@163.com; 3 Jiangsu Leuven Instruments Corporation, Xuzhou 221000, China; ts20020126p21@cumt.edu.cn

**Keywords:** measurement while drilling, stratum interface, drilling specific energy, PSO-LF, mine roadway support

## Abstract

The precise detection of stratum interfaces holds significant importance in geological discontinuity recognition and roadway support optimization. In this study, the model for locating rock interfaces through change point detection was proposed, and a drilling test on composite strength mortar specimens was conducted. With the logistic function and the particle swarm optimization algorithm, the drilling specific energy was modulated to detect the stratum interface. The results indicate that the drilling specific energy after the modulation of the logistic function showed a good anti-interference quality under stable drilling and sensitivity under interface drilling, and its average recognition error was 2.83 mm, which was lower than the error of 6.56 mm before modulation. The particle swarm optimization algorithm facilitated the adaptive matching of drive parameters to drilling data features, yielding a substantial 50.88% decrease in the recognition error rate. This study contributes to enhancing the perception accuracy of stratum interfaces and eliminating the potential danger of roof collapse.

## 1. Introduction

Roof accidents have remained a significant safety concern in the coal-mining industry. In 2022 alone, there were 31 fatal roof accidents reported in Chinese coal mines, resulting in 66 fatalities. These incidents accounted for approximately 18.5% of the total number of coal mine accidents and 26.9% of the overall deaths. There is always a sudden and violent collapse of the roadway roof in a roof accident, accompanied by the release of extensive contained energy, and the collapse of roof rocks into the roadway [1]. Most roof accidents can be attributed to the delayed detection of geological changes within the roof rock layer [2]. This delay primarily stems from the absence of real-time monitoring capabilities for obtaining crucial interface information using conventional physical exploration methods in coal mines [3]. The properties of interfaces in composite rock layers include crucial information such as structural surfaces, slip surfaces, and material differentiation surfaces, which are of great significance for reflecting key structural factors in roof support design, such as cracks, weak interlayers, and key layers [4]. Hence, the real-time detection of roof interfaces in the drilling process of roadway anchoring holes holds great significance. It enables a timely reflection of geological changes in roof layers, the optimization of support parameters, and the proactive mitigation of potential roof collapses.

To achieve the real-time sensing of interface positions during rock formation drilling, numerous scholars have conducted extensive research on feature indicators and recognition algorithms for interface positioning using drilling techniques [5].

In the domain of interface characteristic indicators, several researchers have made notable contributions. Itakura et al. [6] developed a real-time data acquisition measurement-while-drilling (MWD) system and employed the torque, thrust, rotational speed, and displacement analysis to identify discontinuous surfaces within rock formations during drilling processes. Chen et al. [7] explored the response characteristics of hydraulic pressure and flow rate in anchor drilling rig systems during roof drilling. They proposed that a step or impulse response in these parameters could indicate passage through roof interfaces or weak interlayers. Raymond Leung et al. [8], on the other hand, focused on modulating drilling power and energy indexes while constructing modulated specific energy (SEM) indexes with stage separation characteristics. Through methods such as mutual information analysis, simple threshold strategies, and artificial neural networks, they successfully verified the high sensitivity of SEMs in distinguishing coal/non-coal interfaces. Liu et al. [9,10], meanwhile, conducted a comparative analysis of different rock strengths’ effects on distribution patterns observed in drilling speed, sound pressure levels, and drill cutting sizes. Their results indicated that, under effective noise removal using MATLAB sine wave models for data-processing purposes, the drilling speed exhibited superior interface recognition effectiveness compared to sound pressure levels; the drill cutting size showed a comparatively lesser efficacy.

In terms of interface recognition algorithms, Labelle et al. [11] conducted on-site roof-drilling data collection and employed machine learning techniques for feature recognition. This approach enabled the approximate classification of coal–rock interfaces, interbedded layers, and other formations. Rostami et al. [12] performed indoor experiments and developed a fissure detection program based on the cumulative sum (CUSUM) algorithm using thrust and composite indexes. This method successfully identified fissures wider than 2 mm. Liu et al. [9] utilized both the K-means clustering algorithm and change point detection (CPD) algorithm to process drilling data from composite strength mortar specimens. They discovered that the drilling speed exhibited good responsiveness when crossing interfaces, while the CPD algorithm demonstrated excellent anti-interference capabilities. Chen et al. [13], on the other hand, established a database of high-resolution images, encompassing limestone, dolomite, loess clay, and red clay samples. They proposed a high-precision rock-type classification method employing deep convolutional neural networks for image recognition in conjunction with deep learning techniques. This approach achieved the high-precision detection and quantification of various rock interfaces.

The aforementioned studies demonstrate that intelligent algorithms can be utilized to monitor and process index data collected during roof-drilling processes for the detection and localization of rock interfaces. However, the current research still faces several challenges:

(1) Many studies rely on direct drilling indicators or strength characteristic indicators for interface detection, such as the thrust, torque, and drilling specific energy (SED). While these indicators primarily showcase the state distribution of the drilling process or highlight differences in rock strength characteristics, they often lack sensitivity toward interfaces. This can lead to misleading detection results, due to either exaggerated features in non-interface stages or subtle features in interface stages.

(2) Various types of intelligent algorithms have been applied to interface detection. However, most applications mainly focus on directly realizing interface recognition through a certain algorithmic function. The optimization research based on the interface recognition principles and characteristics of drilling data is not as prolific as the former, resulting in limited recognition accuracy and generalizability in the model.

Most applications mainly focus on directly realizing interface recognition through a certain algorithmic function, such as Liu’s monitoring of drilling-speed data for interface localization through the CPD algorithm. Targeted optimization based on the interface recognition principle and characteristics of drilling data and other characteristics of the research problem is limited, resulting in the model not reaching a high recognition accuracy.

In this study, based on the principle of interface detection in rock strata through change point detection, the detection capability of the SED was reinforced by the logistic function. And the driving parameters of the model were modulated with the particle swarm optimization algorithm, through leveraging the drilling data characteristics. Consequently, the precise detection and localization of interfaces was accomplished under small displacements.

## 2. Model

In the detection study for rock interfaces, it is commonly assumed that the uniaxial compressive strength (UCS) of the rock and the drilling parameters remain stable during non-interface stages, while the UCS exhibits changes at the interface [14]. Consequently, the drilling state of any interface position during the drilling process can be expressed as Equation (1).

(1) $$C=\frac{\sigma_{c}^{1}-\sigma_{c}^{2}}{L}=\frac{K(I_{d}^{1}-I_{d}^{2})}{L}$$

where C represents the characteristic value of the interface, $\sigma_{c}^{1},\sigma_{c}^{2}\;$ correspond to the UCS on both sides of the intersection, L represents the length of the interface, which is the thickness of the rock cementation area in practice, $I_{d}^{1},\;I_{d}^{2}$ represent the corresponding values of the drilling index for the two rock properties, and K signifies the correlation coefficient between the rock properties and drilling indices.

To account for the typically small length of the interface, the characteristic value C can be approximated as the derivative of the rock properties at the interface, which can be expressed as Equation (2).

(2) $$C=\underset{L\to 0}{\lim}\frac{\sigma_{c}^{1}-\sigma_{c}^{2}}{L}=\underset{L\to 0}{\lim}\frac{K(I_{d}^{1}-I_{d}^{2})}{L}=P^{\prime }=KI_{d}^{\prime }$$

Assuming that the changing rate of drilling index $I_{d}^{\prime }$ for the rock partition interface is R, the judgment criteria for rock interfaces at location h can be defined as Equation (3).

(3) C(h)≥KR

From the above analysis, it can be feasible to position rock interfaces by monitoring the change point of the drilling index data. When the rate of change of the drilling index exceeds KR at a certain point, it means that the drill bit is crossing the rock-parting interface, where the corresponding location is h.

**Figure 1:**
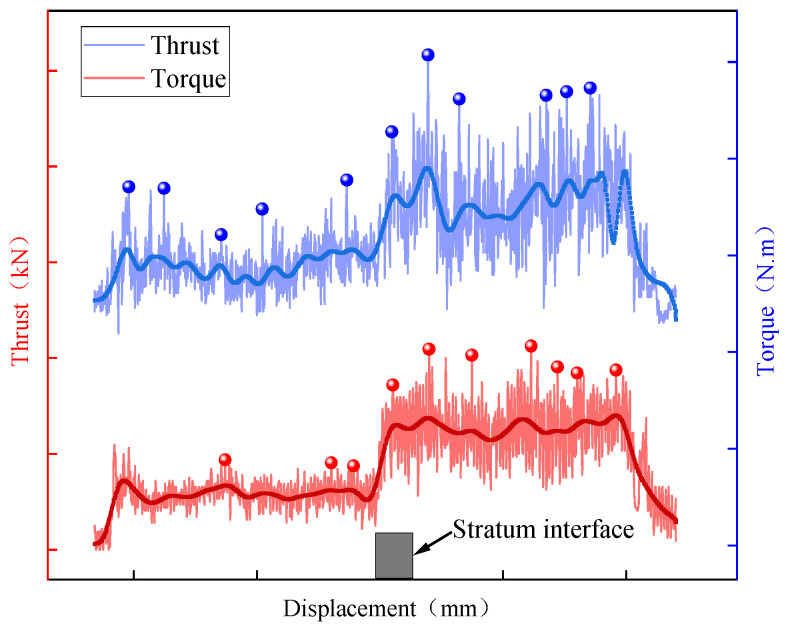
The displacement distribution of the drilling parameters.

However, during the actual drilling process, various disturbances, such as non-homogeneous bodies and equipment limitations, exist, leading to fluctuations in the collected data [15], as illustrated in Figure 1. The local peak points on the curve are labeled, and it can be observed that peak points with high rates of change can interfere with the accurate detection and positioning of interfaces. This interference is particularly significant for indicators with a high volatility, such as the torque, as the rate of change of certain peak points on the curve surpasses the corresponding characteristic value of the interface stage points, resulting in recognition errors.

**Figure 2:**
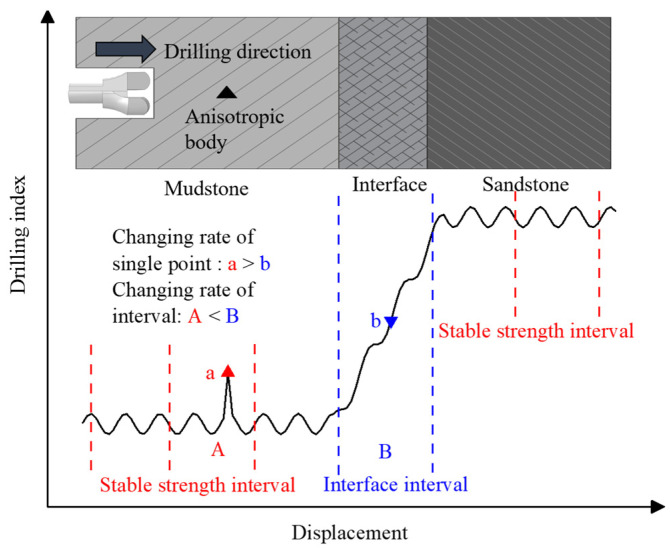
The effect of data intervals on the drilling characteristics of composite formations.

Due to the susceptibility of individual data to disturbances, a more practical approach to reflecting the rate of change of indicators is to utilize the coefficient of variation for data intervals [16]. Figure 2 demonstrates the division of the drilling data into intervals of a specific length. Despite the presence of a disrupted mutation point, ‘a’, in the stable intensity stage, caused by external factors like non-homogeneous bodies, the degree of change within the interval remains relatively low, thereby not affecting the detection of the interface interval. The coefficient of variation serves as a dimensionless measure reflecting the degree of data fluctuation, which can be expressed as Equation (4).

(4) $$c_{j}^{}=\frac{\sigma_{j}^{}}{{\bar{I}}_{j}}=\sqrt{\frac{l\times {\bar{I}}_{j}{}^{2}}{\sum_{i=1}^{l}{\left(I_{j}^{i}-{\bar{I}}_{j}\right)}^{2}}}$$

where $c_{j}$, $\sigma_{j}$, ${\overset{¯}{I}}_{j}$ are the coefficient of variation, the standard deviation, and the mean of the *j*-th drilling interval. $I_{j}^{i}$ is the *i*-th datum of the drilling interval. And l is the length of the interval. Moreover, to ensure the consistency of the corresponding displacement lengths of intervals, the data lengths of the intervals at the three penetration speeds were 16, 10, and 8, respectively.

In a word, building upon the preceding analysis of drilling interface characteristics and detection feasibility, a method for detecting interfaces by monitoring the change point of the interval coefficient of variation using the cumulative sum (CUSUM) algorithm is proposed. In the drilling of non-interfacial rock formations, the lithology tends to remain relatively stable, resulting in minimal fluctuations in the corresponding interval coefficient of variation of the drilling index. However, when the drill bit encounters the interface between rock formations, the lithology on either side of the interface changes, leading to a noticeable fluctuation in the interval coefficient of variation.

By employing the CUSUM algorithm, the proposed method effectively tracks and identifies significant changes in the interval coefficient of variation of drilling indexes, enabling accurate interface positioning [17]. The main steps to interface detection through the change point for the drilling indicators are as follows:

① Calculate the coefficient of variation for each interval c, and initialize the basic parameters of the CUSUM algorithm, such as the system minimum error e and the initial value of the cumulative sum $s_{0}$.

② Calculate the cumulative sum $s_{j}$ corresponding to the *j*-th data point $c_{j}$, which is defined in Equation (5).

(5) $$s_{j}=s_{j-1}+c_{j}-\bar{c}-e$$

where $s_{j}$ represents the cumulative sum of the deviations from the mean for the *j*-th datum and all previous data, and $s_{+}$ and $s_{-}$ are the maximum and minimum values, respectively.

③ Calculate the monitoring threshold $h_{j}$ corresponding to the data point $c_{j}$, which is defined in Equation (6).

(6) $$\left\{ \begin{array}{l} h_{j}^{}=k\sigma_{j}^{*}\\ \sigma_{j}^{*}=\sqrt{\frac{\sum_{i=1}^{j}{\left(c_{i}-\bar{c}\right)}^{2}}{j}} \end{array}\right.$$

where k is the correlation characteristic parameter between the CUSUM model monitoring threshold and the interval coefficient of variation. It is set to 4 in this paper, which is proven to have a good balance between monitoring effectiveness and immunity to interference after a large number of practical applications of the CUSUM algorithm. And $\sigma_{j}^{*}$ is the standard deviation of the dataset consisting of the point $c_{j}$ and the data before that point.

④ When $s_{j}$ meets the conditions in Equation (7), the interval fluctuation degree is considered to exceed the threshold value, the data are marked as an interface feature point, and the value is outputted with the corresponding interval sequence number (*j*, $s_{j}$).

(7) $$\left\{ \begin{array}{l} s_{+}^{}-s_{j}^{}\geq h_{j}\\ s_{j}^{}-s_{-}^{}\leq h_{j} \end{array}\right.$$

The former applies to the “high strength to low strength” drilling sequence test, while the latter applies to the “low strength to high strength” drilling sequence test.

**Figure 3:**
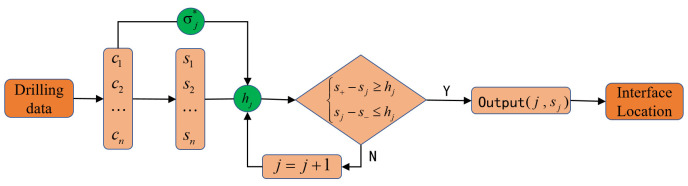
Flowchart of the CUSUM model.

Figure 3 shows the flow diagram of the CUSUM model.

## 3. Experimental Device and Materials

### 3.1. Test System and Materials

**Figure 4:**
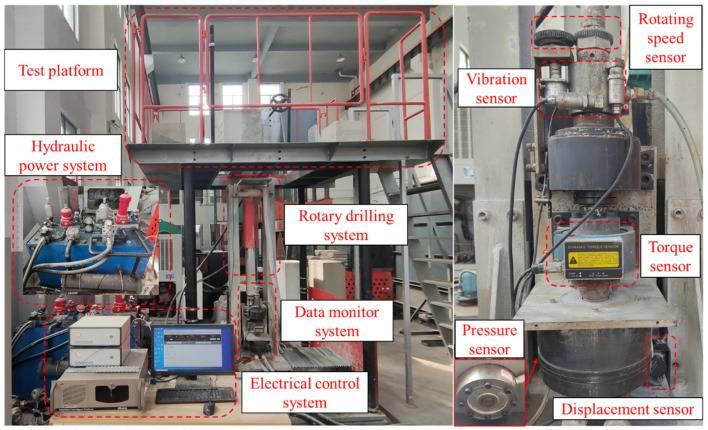
The drilling test systems and the sensors.

The drilling test system used for this drilling test is shown in Figure 4. The test system consists of a test platform, hydraulic power system, electrical control system, rotary drilling system, and data monitoring system. To be more specific, the hydraulic power system can provide a maximum torque of 400 N.m, a maximum thrust of 100 kN, and a propulsion stroke of 1000 mm. The electrical control system incorporates control devices and a computer, enabling a precise control over the rotational and penetration speeds. Moreover, the data monitoring system consists of a programmable logic controller (PLC) and high-precision sensors, allowing for real-time monitoring and data collection concerning various parameters such as the thrust, torque, displacement, rotational speed, and vibration, with a frequency of 3 Hz. In the conducted tests, standard B19 drill pipes and a Φ28 PDC drill bit were utilized for mining purposes.

In this test, three mortar samples were prepared to simulate drilling in composite-strength rock formations with different lithologies. The dimensions of the mortar samples were 300 mm × 300 mm × 300 mm, and they were designed to have specific uniaxial compressive strength (UCS) types: 20 MPa × 40 MPa, 30 MPa × 50 MPa, and 30 MPa × 40 MPa. Table 1 presents the strengths of the samples after maintenance and the locations of the partition interfaces.

**Figure 5:**
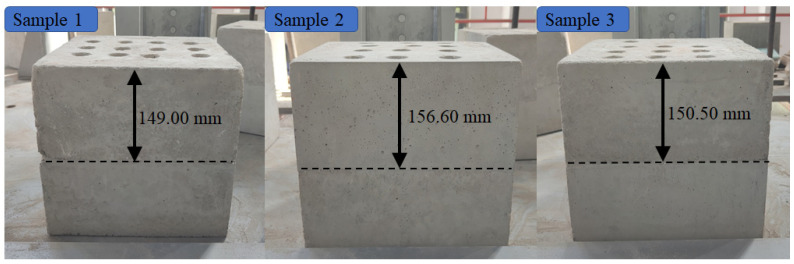
Mortar samples after drilling.

Following the stability test for the drilling process, the penetration speeds of 0.5 mm/s, 0.8 mm/s, and 1 mm/s were carefully chosen to investigate rock interface detection under various penetration speeds. The rotation speed of 450 rpm and the drilling displacement of 260 mm were maintained throughout the tests. To minimize data interference from external factors, three holes were drilled at each penetration speed, and the mean values were ultimately selected as the final test data. The mortar samples after drilling are shown in Figure 5.

### 3.2. Data Analysis

#### 3.2.1. Characteristics of Drilling Parameters

The samples were drilled based on the test protocol, and data on the thrust, torque, displacement, rotating speed, and displacement were collected for the whole drilling test. To analyze the data characteristics of the drilling parameters, a representative test group, denoted as 1-450-0.8, was selected for in-depth analysis.

**Figure 6:**
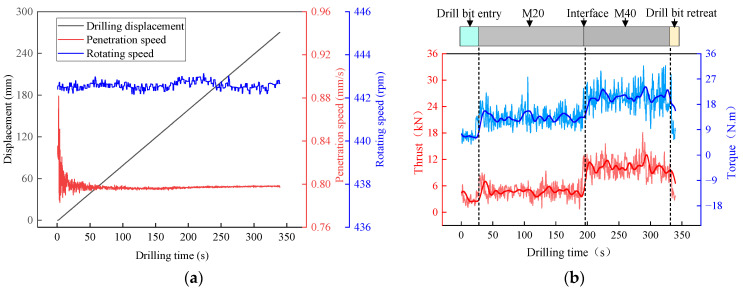
(**a**) The drilling-parameter–time curve, (**b**) the distribution of the thrust and torque.

Figure 6a shows the time characteristic curves of penetration speed, rotating speed, and displacement during the drilling process. The curves revealed that the rotating speed consistently maintained stability within the range from 442 rpm to 444 rpm. The penetration speed, after experiencing some initial fluctuations, settled at an average of approximately 0.8 mm/s. Furthermore, the slope of the drilling displacement curve remained relatively constant. The above analysis verifies the great control effect on the rotating speed and penetration speed of the test system.

Figure 6b illustrates the curves depicting the changes in the thrust and torque over time during the drilling process. It is evident that the thrust and torque exhibit distinct distribution patterns at different intensities. However, direct drilling indicators such as the thrust and the torque display an overall data volatility, due to the influence of external factors, which results in a multi-peak distribution. Furthermore, it is important to note that the drilling data exhibited abnormal fluctuations at the beginning and end of the drilling process, due to the “drill-in” and “drill-out” operations. These fluctuations significantly deviated from the general distribution range. So, it is necessary to exclude such corresponding invalid data from subsequent data analysis.

#### 3.2.2. Interface Recognition Performance of SED

**Figure 7:**
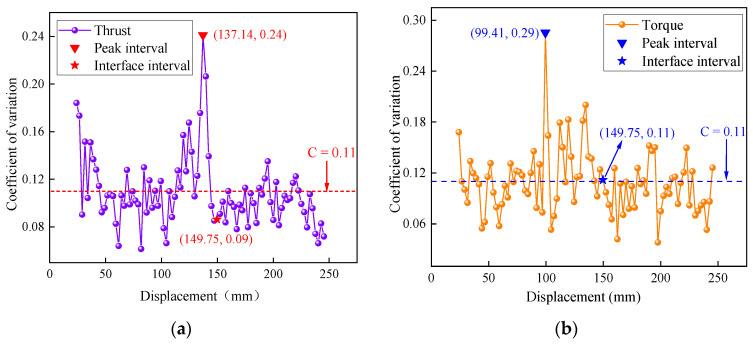
(**a**) The distribution of the thrust intervals, (**b**) the distribution of the torque intervals.

To evaluate the feasibility of interface detection using the thrust and torque, an analysis of the distribution characteristics of interval fluctuations for these indicators was conducted. Figure 7 present the distribution curves of the variation coefficients for the intervals of thrust and torque, focusing on the typical test group 1-450-1.0.

Firstly, it can be observed that the average interval variation coefficient for both the thrust and the torque was 0.11, indicating a high degree of fluctuation. The coordinates of the peak points and interface corresponding points for the two indicators were (137.14, 0.24), (149.75, 0.09), and (99.41, 0.29), (149.75, 0.11), respectively. Notably, the peak points of the direct indicators appeared to be significantly distant from those in the interface stage. Particularly for the torque, there was a displacement deviation of approximately 50 mm. This suggests that the thrust and torque were prone to disturbances by external factors, leading to substantial fluctuations even during stable drilling stages.

Secondly, the interval variation coefficients of the indicators at the interface were relatively small and significantly differed from the corresponding peak points. This implies that the indicators did not exhibit a strong volatility when the drilling intensity changed, indicating a low sensitivity to changes in the interface structure. In summary, the thrust and torque demonstrate a limited resistance to interference and a low sensitivity to interfaces, resulting in a low level of compatibility with the CUSUM interface detection model.

As direct indicators such as the thrust and torque have a low adaptability, the CUSUM interface detection model of integrated drilling indices is more strongly recommended for interface detection in this study. There is quite a lot of research on integrated indicators in the field of measurement while drilling. The rock-drilling specific energy indicator SED, proposed by Teale R, is chosen for the study, and is expressed in Equation (8) [18]. This indicator provides a clear definition of the drilling specific energy consumption and has a simple structure [19]. Moreover, the SED demonstrates a notable specificity within the drilling process for different rock strengths, meaning that it is widely recognized in the field of rock strength detection while drilling.

(8) $$SED=(W_{n}+W_{t})/V=(\tau \omega +F_{n}v_{n})/\pi r^{2}v_{n}$$

where τ is the torque, ω is the rotating speed, $F_{n}$ is the drilling thrust, $v_{n}$ is the penetration speed, and r is the drilling radius.

However, while the effectiveness of the SED has been acknowledged for rock strength detection, its compatibility with the CUSUM model for interface detection still requires investigation. Therefore, it is imperative to evaluate the feasibility of the SED for interface detection.

**Figure 8:**
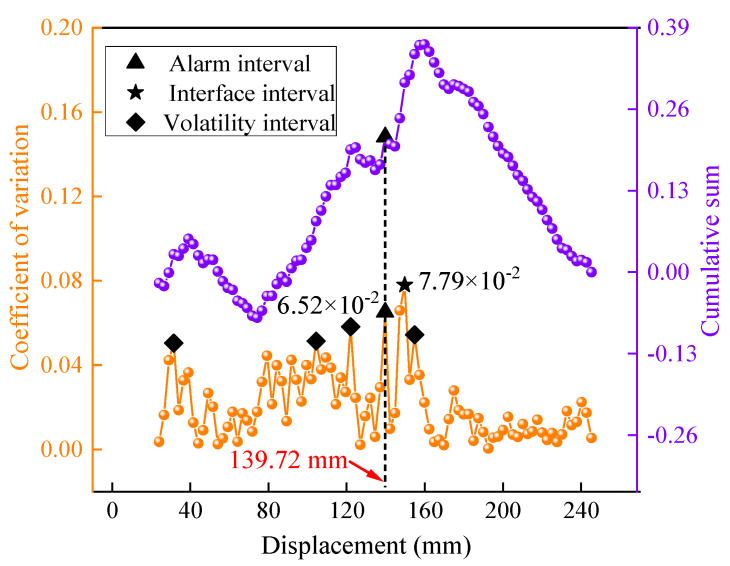
The recognition effect of the SED.

Figure 8 illustrates the recognition effect for test group 1-450-1.0. The lower curve represents the distribution curve of the interval variation coefficient for the SED index, while the upper curve displays the distribution curve of the interval cumulative sum. Upon analyzing the coefficient of variation distribution curve, it was evident that the mean value of the interval fluctuation degree for the SED was 2.21 × 10^−2^, which was significantly lower than that of the thrust and torque. In contrast, the interval variation coefficient for the interface was 7.79 × 10^−2^, notably higher than the average. This indicated that the SED exhibited a higher sensitivity to drilling interfaces compared to the thrust and torque. From the cumulative sum curve distribution, the SED had an alarm indication at the drilling displacement of 139.72 mm. And the interface detection error was 9.28 mm, primarily stemming from the presence of numerous points with high fluctuations in the SED variation coefficient curve. The sudden changes in the cumulative sum values corresponding to these fluctuations interfered with the ability to accurately identify interface positions.

## 4. Method

### 4.1. Enhancing Interface-Recognition Ability of SED through Logistic Function

Based on the aforementioned analysis, it can be concluded that the SED indicator exhibits a better specificity in distinguishing between the drilling interface stage and the non-interface stage, compared to direct indicators such as the thrust and torque. However, the presence of a significant number of interfering points in its variation coefficient curve diminishes its effectiveness in highlighting the interface feature points. Consequently, this leads to suboptimal detection results. So, the SED should be optimized from two aspects: (1) to reduce the drilling volatility of the indicator in the non-interface stage, leading to a decline in the interference of anomalies for monitoring and positioning feature points; (2) to improve the sensitivity of the indicator when drilling through interfaces, to enhance the “highlighting” effect of interface feature points compared with other drilling stages, to achieve a better detection and positioning effect.

Based on the aforementioned optimization proposal, the application of a logistic function is suggested to modulate the SED indicator to enhance its effectiveness in recognizing interfaces [20]. So, the specific energy of the logistic function (*SEL*) is defined as the composite form of the logistic function and power function of the SED, which is expressed as Equation (9).

(9) $$\left\{ \begin{array}{l} SEL={\left\{\frac{b}{1+\exp\left[-a(E-c)\right]}+g\right\}}^{N}\\ E_{n}=uSED_{n}+(1-u)E_{n-1} \end{array}\right.$$

In Equation (9), the logistic function can separate the non-interface stages and interface stages in drilling, to extract the difference characteristics of various drilling stages. N can amplify the difference characteristics of two stages and improve the specificity of the indices, which is set to 2. The core parameters a and c control the width and overall direction of the conversion stage of the sigmoid curve and conversion point in the logistic function, respectively, which can directly affect the separation effect of indicators on different stages of drilling [21]. According to the characteristics of the drilling data of sample no. 1, the values of a and c are set as 1.20 and 1.37, respectively. The parameters b and g regulate the range of index values to avoid extreme values that can affect the interface detection effect, and they are set as 1 and 0.4, respectively. Moreover, the formula between $E_{n}$ and $SED_{n}$ is a method of exponential smoothing. It can reduce the data noise and variance in the SED, leading to a decrease in data volatility. And n represents the *n*-th data and u is set as 0.6 to achieve a good balance between the original level of data distribution and the data-smoothing effect.

### 4.2. Effect of Driving Parameters on the Logistic Function

The fundamental principle of the *SEL* is to distinguish the stable drilling stage from the interface stage by matching the conversion stage of a logistic curve within its internal structure with the interval of the interface characteristic data. To investigate the interface detection effect under different matching states of the logistic curve transition stage and interface feature interval, a logistic-type index L(x) is formulated as Equation (10). The optimal values of parameters a and c, corresponding to interface features, are determined as 1 and 1.5, respectively. When the parameters are taken as the ideal values, it means that the characteristic parameters of the logistic indexes are consistent with the interface characteristic values of the drilling data. This means that the positions of the logistic curve conversion stage and the interface interval of the drilling data are consistent. On the basis of not changing the interface characteristics of the drilling data themselves, it has a good effect on sharpening the different characteristics of the different drilling stages.

(10) $$L(x)=\frac{0.5}{1+\exp\left(-a(x-c)\right)}+1$$

#### 4.2.1. Effect of Driving Parameter a on the Logistic Function

To study the impact of the characteristic parameter a on the logistic-type indicators, different parameter values were applied to the indicator L(x) function model, and their corresponding effects were compared and analyzed.

**Figure 9:**
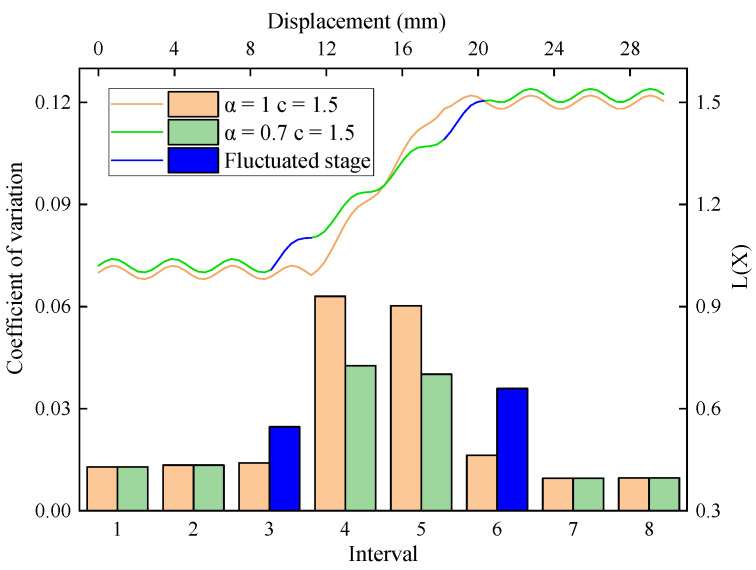
L(x) function model when c=1.5 and a are taken as 0.7 and 1.0.

Figure 9 shows the distribution of the L(x) function and interval variation coefficients when c=1.5 and a are taken as 0.7 and 1.0, which correspond to the actual value and ideal value of the parameter, respectively. The transition phase from the low to high levels of the curve was longer at 0.7 than at 1.0. Additionally, the former exhibited a wider distribution of intervals with high variation coefficients and less pronounced interval peaks. It shows that, when the actual value of the parameter is lower than the ideal value, some data at the periphery of the interface stage would fluctuate. Consequently, the variation coefficient of the corresponding interval rises, leading to a higher proportion of fluctuating intervals. These fluctuations can significantly interfere with the accuracy of the recognition results.

**Figure 10:**
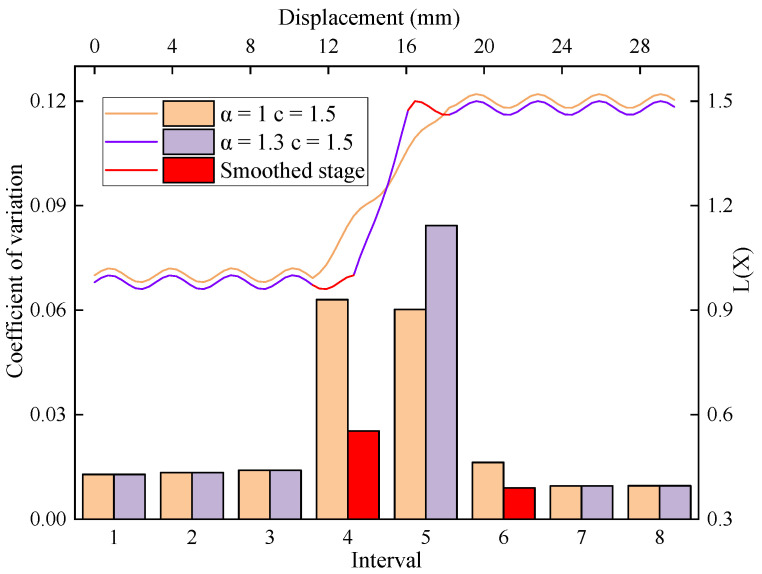
The L(x) function model when c=1.5 and a are taken as 1.3 and 1.0.

Figure 10 shows the distribution of the L(x) function and the interval coefficients of variation when c=1.5 and a are taken as 1.3 and 1.0, corresponding to the actual and ideal values of the parameters, respectively. The transition phase from the low to high levels of the curve was shorter for 1.3 compared to 1.0. Additionally, the former exhibited a narrower distribution of intervals with high variation coefficients. When the actual value of the parameter exceeds the ideal value, it results in the smoothing of some data points within the interface stage and a reduction in the coefficients of variation for the corresponding intervals. Consequently, there is a risk of excluding the actual interface interval from identifiable intervals.

Comparing the effects of the L(x) function under the three cases of large, optimal, and small values of a, the value of a significantly affects the length of the transformation stage of the L(x) function curve. A larger or smaller value of the parameter can result in significant changes in the nature of some drilling data within the interface stage, as well as a low degree of matching between the logistic curve structure and the actual data features, which might adversely affect the recognition effect of the index.

#### 4.2.2. Effect of Driving Parameter c on the Logistic Function

To study the impact of parameter c on the logistic-type indicators, different values of the parameter were applied to the indicator L(x) function model, and their expression effects were compared and analyzed.

**Figure 11:**
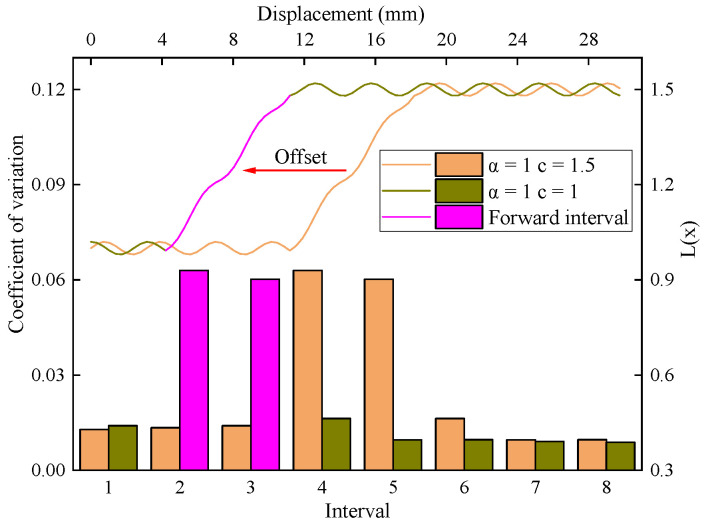
The L(x) function model when a =1.0 and c are taken as 1.0 and 1.5.

Figure 11 shows the distribution of L(x) function values and interval variation coefficients when a =1.0 and c are taken as 1.0 and 1.5, which correspond to the actual value and ideal value of the driving parameters, respectively. The curves corresponding to the intervals of No. 2 and No. 3 exhibited high fluctuations at c=1.0. Additionally, the curves corresponding to the intervals of No. 4 and No. 5 demonstrated high fluctuations at c=1.5. These observations indicated that, compared to the latter, the entire conversion phase of the former was shifted forward. It means that, when the actual value of parameter c is smaller than the ideal value, the interface interval will be misaligned, and its position will be shifted to the front of the actual interface position, confusing the interface detection.

**Figure 12:**
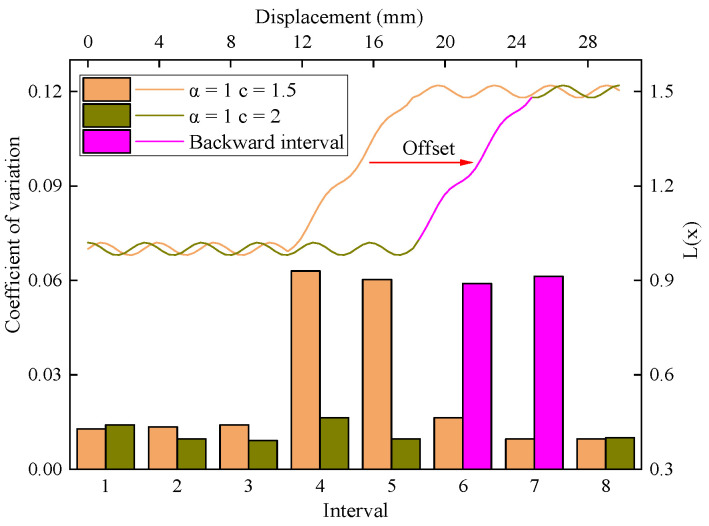
The L(x) function model when a=1.0 and c are taken as 1.5 and 2.

Figure 12 shows the distribution of the L(x) function and the interval variation coefficients when a =1.0 and c are taken as 1.5 and 2, corresponding to the actual value and the ideal value of the driving parameters, respectively. The high fluctuation intervals corresponding to the curves at 1.5 and 2 were No. 4, No. 5 and No. 6, No. 7, respectively, which meant that, compared with the former, the conversion phase of the latter was shifted backward. Correspondingly, if the actual value of parameter c is larger than the ideal value, the interface feature intervals will be backwardly misaligned, and their corresponding position will be shifted to the back of the actual interface position, which could interfere with the detection and positioning of interface features.

By comparing the effects of the L(x) function under three scenarios, with large, optimal, and small values of c, it is evident that the parameter value significantly influences the position of the transition phase in the L(x) function curve. A larger or smaller value of c can cause a deviation in the drilling feature phase, leading to a reduced degree of matching between the logistic curve structure and the actual data features, which could have a direct impact on the recognition effectiveness of the index.

The above study describes the varying matching conditions and their effects on recognition errors that arise from the differences between the actual values and ideal values of the parameters a and c. Therefore, in the context of the *SEL*, the values of these driving parameters significantly impact the matching conditions between the logistic curve transformation stage and the interface feature interval. So, it is concluded that parameters a and c play a crucial role in determining the accuracy of the interface recognition results. In other words, improper values will cause a significant increase in errors. Therefore, it is necessary to conduct optimization research on the values of a and c to mitigate recognition errors.

### 4.3. Self-Adaptive Optimization of Driving Parameters through PSO Algorithm

To achieve a high accuracy of interface detection for *SEL* indicators under different drilling conditions, a study on the self-adaptive optimization of driving parameters a and c based on the particle swarm optimization (PSO) algorithm was carried out. 

The PSO algorithm is one of the most widely used intelligent optimization algorithms with a good optimization effect, and is inspired by the foraging activities of birds. The principle is that each individual in a group of birds updates its next foraging position based on its optimal position and the collective optimal foraging position of the group, until the optimal position in the territory is discovered [22]. The iterative update rules for the particle’s flight speed v and position x are presented in Equations (11) and (12).

(11) $$v_{id}(t+1)=wv_{id}(t)+c_{1}rand_{1}(p_{id}(t)-x_{id}(t))+c_{2}rand_{2}(p_{gd}(t)-x_{id}(t))$$

(12) $$x_{id}(t+1)=x_{id}(t)+v_{id}(t+1)$$

where *t* is the number of iterations; d=1, 2, ⋯ ; D is the dimension of the search space, i=1, 2, ⋅⋅⋅ ; m is the number of particles; $v_{id}\left(t\right)$ $v_{id}\left(t\right)$ is the flight speed of the *i*-th particle in *d*-dimensional space; $c_{1}$ and $c_{2}$ are the individual learning factor and the population learning factor; $rand_{1}\left(\;\right)$ and $rand_{2}\left(\;\right)$ are random numbers uniformly distributed on the interval [0, 1]; $p_{id}\left(t\right)$ is the historical optimal position of the particle at the *t*-th iteration; $p_{gd}\left(t\right)$ is the historical optimal position of the population at the *t*-th iteration; and $x_{id}$ is the position of the *i*-th particle in *d*-dimensional space.

The fitness function, which represents the foraging effect, plays a crucial role in assessing the effectiveness of the PSO algorithm [23]. To achieve a desirable separation effect of different drilling stages for the *SEL*, the fitness function f(x) for the *SEL* optimization model is defined as Equation (13).

(13) $$\left\{ \begin{array}{l} f(x)=\sum_{1}^{m}\left|Q(i)-S(i)\right|\\ q(i)=\frac{1}{1+\exp\left(-x_{1}\times (s(i)-x_{2})\right)}+0.4\\ \left[Q(i),S(i)]=[\frac{q(i)-q{\left(i\right)}_{\min}}{q{\left(i\right)}_{\max}-q{\left(i\right)}_{\min}},\frac{s(i)-s{\left(i\right)}_{\min}}{s{\left(i\right)}_{\max}-s{\left(i\right)}_{\min}}\right] \end{array}\right.$$

where $x_{1}$ and $x_{2}$ correspond to the driving parameters a and c, respectively. s(i) is the *i*-th datum of the SED indicator, m is the amount of data of the SED indicator, and f(x) has a negative correlation with the separation effect of the *SEL* drilling stages.

**Figure 13:**
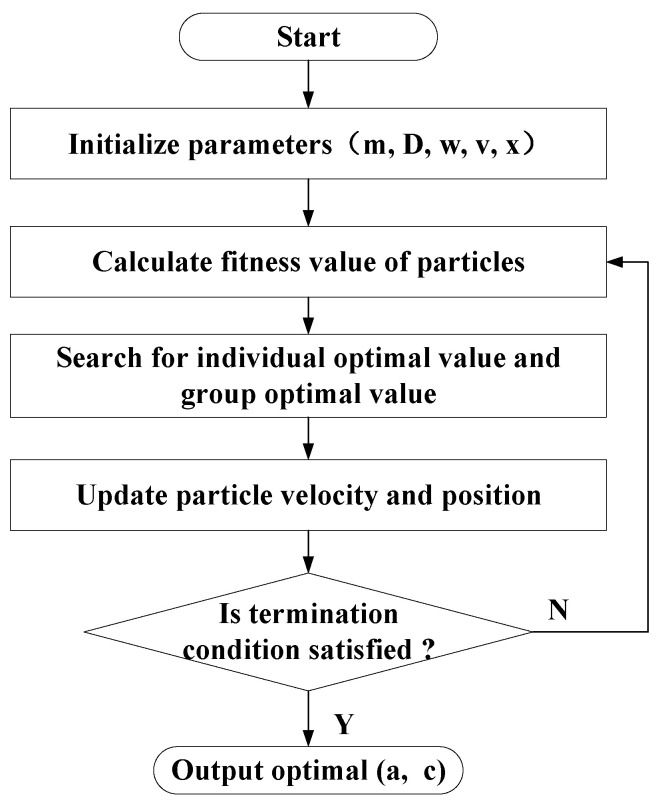
The basic flow of the PSO algorithm.

The PSO optimization model utilizes the fitness function to determine both the individual optimal parameter value and the group optimal parameter value in each generation. Through iterative updates based on optimization rules, the model aims to reach the historical optimal values of a and c, which lead to the best recognition effect for the *SEL*. The flow chart of the PSO algorithm is presented in Figure 13.

## 5. Results

### 5.1. Effectiveness of SED with Logistic Function

**Figure 14:**
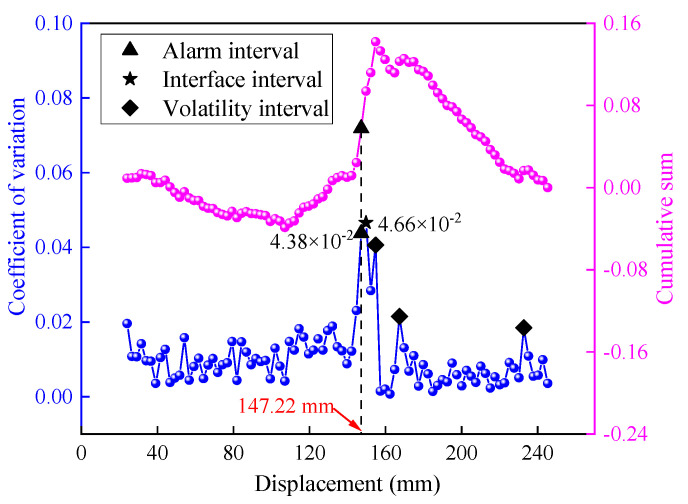
The recognition effect of the *SEL*.

To verify the effectiveness of the logistic function, the *SEL* data corresponding to the test group 1-450-1.0 were incorporated into the detecting model, as depicted in Figure 14.

The *SEL* exhibited an alarm indication at a drilling displacement of 147.22 mm, with an interface detection error of 1.78 mm. A comparison of the detection effects between the two indices is presented in Table 2. When comparing the *SEL* indicator with the SED indicator, there was a notable decrease in the mean value of the interval variation coefficient. Specifically, it decreased from 2.21 × 10^−2^ to 1.04 × 10^−2^, resulting in a significant reduction in the overall fluctuation degree. Furthermore, the number of abnormal fluctuation points decreased from 4 to 3, and their distribution range became more concentrated near the interface position. As a result, the interference from these fluctuation points was minimized. More importantly, the introduction of the *SEL* indicator yielded a substantial improvement in the recognition accuracy. The recognition error was reduced from 9.28 mm to 1.78 mm, demonstrating a significantly higher level of accuracy compared to the SED.

By comparing of the interval coefficient of variation curves for the two indicators, we explored recognition error reduction. During the stable drilling stage, the distribution of the *SEL* interval variation coefficients was more uniform, and the corresponding curve appeared smoother and less prone to fluctuations. In contrast, the SED curve exhibited more high fluctuation points and demonstrated less stability. This stark contrast indicates that the *SEL* indicator is more resilient to disturbances during the non-interface drilling stage, surpassing the performance of the SED. Meanwhile, in the interfacial stage, the interfacial coefficient of variation corresponding to the SED did not exhibit a significant “protrusion” effect compared to the general distribution level, resulting in a limited discriminative capability. Conversely, the interfacial characteristic value of the *SEL* was markedly higher than the general distribution value, indicating a strong sensitivity to interface detection.

Based on the analysis of detection effects, it can be observed that both the *SEL* and the SED demonstrate some level of interface detection ability under specific drilling conditions. However, the logistic function strengthened the immunity under stable drilling and the sensitivity under interface drilling, which led to a more precise interface localization of the *SEL*.

The results for interface recognition are presented in Table 3, revealing notable differences between the *SEL* and SED indicators. The average recognition error for the *SEL* was 2.83 mm, while the SED yielded an average recognition error of 6.56 mm. The recognition accuracy of the *SEL* stood at 56.86%, surpassing that of the SED. These findings validated the enhancement effect of the logistic function for the *SEL*, which achieved a significantly higher recognition accuracy compared to the SED.

### 5.2. Effectiveness of SEL with Self-Adaptive Logistic Function

Due to the mismatch between the drilling data characteristics and the initial values of a and c, the detection errors of samples 2 and 3 were larger compared to those of sample 1. However, considering the similar intensity levels and drilling conditions of the three samples, the overall drilling errors remained within the controllable range. This suggests that the characteristic values corresponding to samples 2 and 3 were within a certain range around the initial parameter values. Consequently, in the subsequent optimization study, the optimization intervals for a and c were set as (0, $3a_{0}$) and (0, $3c_{0}$), respectively, where $a_{0}$ and $c_{0}$ were the initial values of the parameters.

**Figure 15:**
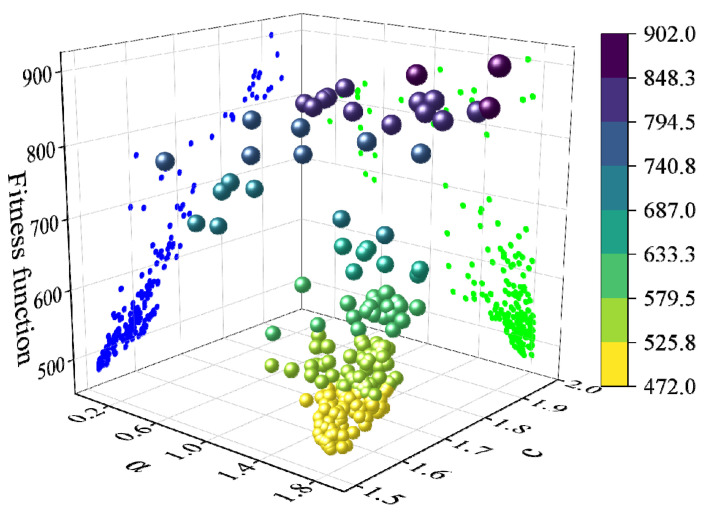
The population particle iterative optimization process.

When establishing a parameter range, the PSO algorithm employs a local search mode, effectively optimizing the search time and minimizing costs in complex conditions. To further illustrate this, group 3-450-1.0 was taken as an example, and Figure 15 shows the iterative particle search process in the corresponding population.

Upon observing Figure 15, it is evident that the particles were initially scattered across the space. Throughout the iterative process, the particles continuously improved their distribution positions based on individual optimal positions and the optimal position of the entire population. Gradually, the particles congregated toward regions with lower fitness values. When a particle reached a position with the lowest fitness, the spatial coordinates (1.61, 1.55) corresponded to the optimal values of the driving parameters a and c. Simultaneously, the optimization iteration for the entire population was also completed.

**Figure 16:**
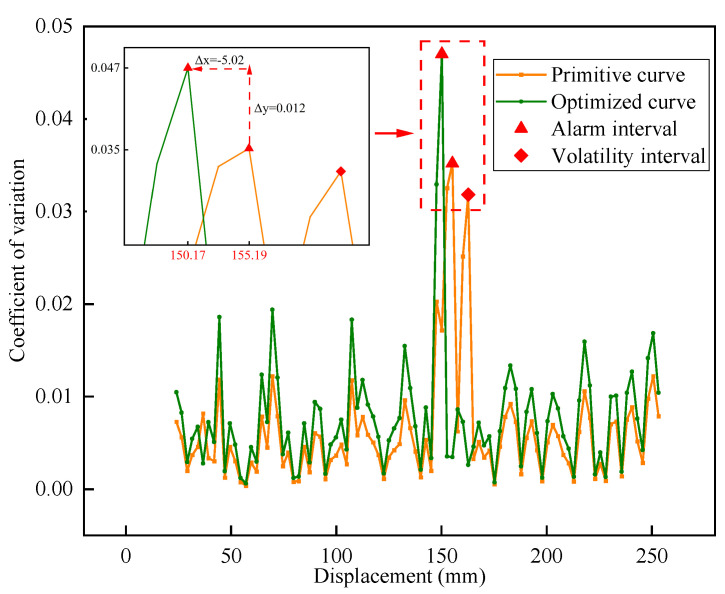
The interval coefficients of variation before and after the optimization of the driving parameters.

In Figure 16, the distribution curve of the interval variation coefficient of the *SEL* index before and after the optimization of the test group 3-450-1.0 is presented. Before optimization, the curve displayed two fluctuating peaks, which could potentially interfere with interface detection. Additionally, the alarm value and corresponding displacement were recorded as 0.035 and 155.19 mm, respectively. However, after optimization, the number of peak points in the coefficient of variation curve reduced from 2 to 1, and the peak value increased by 0.012. Comparatively, the peak became more prominent to the average distribution level, thereby reducing the difficulty of interface detection. Consequently, the detection error was significantly reduced from 4.69 mm to 0.33 mm, and the optimization rate reached an impressive 92.96%. These results demonstrated the substantial optimization and enhancement effects of the self-adaptive logistic function.

The optimization for the *SEL* based on the PSO algorithm achieved the adaptive matching of driving parameters with drilling data features. The optimized values of parameters reflect two specific properties of the data: a represents the inverse of the length of the transition interval between two drilling strengths, and c represents the feature value of the SED when the drill bit contacts the interface.

**Figure 17:**
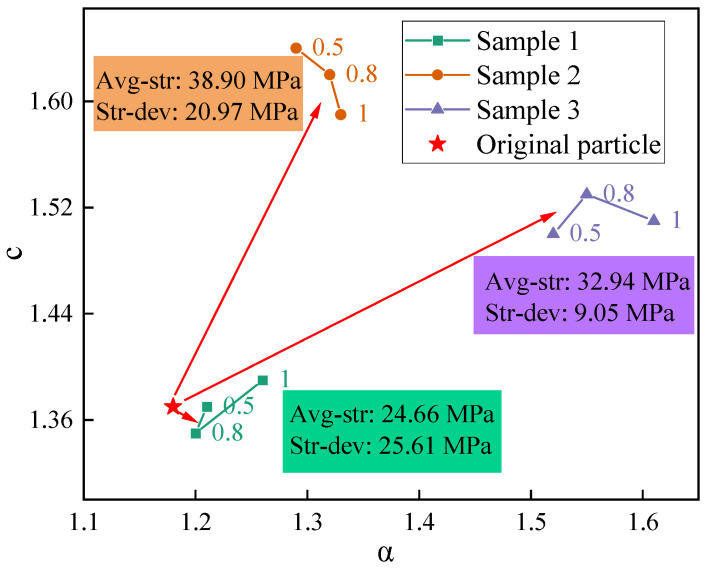
The parameters before and after optimization.

The values of a and c before and after optimization are illustrated in Figure 17. It can be concluded that there is a negative correlation between the sample strength differences and the value of a, while the average strength of the samples shows a positive correlation with the value of c. Additionally, changes in the penetration speed influence the values of a and c. So, it is easy to deduce that an increase in the intersection strength difference increases the length of the transition interval, while an increase in the average strength of the sample leads to an increase in the SED characteristic value when the drill bit contacts the interface. Additionally, changes in the penetration speed impact the data characteristics, thereby causing variations in the length of the transition interval and the SED characteristic value.

The recognition effect of the optimized driving parameters is presented in Table 4. It is evident that the recognition errors significantly decreased before and after optimization, with average error values of 2.83 mm and 1.39 mm, respectively. This corresponds to a remarkable reduction of 50.88% in the error rate. Most notably, the recognition errors for samples 2 and 3 were effectively controlled and no longer exhibited large-scale errors, which greatly enhanced the credibility and stability of interface recognition for the *SEL*.

Based on the above study, it is evident that optimizing the matching degree between the driving parameters and data characteristics can improve the detection precision of the interface, which is supported by the reduction in the mean value of interface detection errors from 2.83 mm to 1.39 mm. This outcome verifies high-precision interface detection under a small drilling displacement, and the significant error optimization effect of the self-adaptive logistic function.

## 6. Discussion

A high-precision rock interface detection model with an average error of 1.39 mm is proposed in this paper, which benefits from two main aspects:(1) the *SEL* with the logistic function exhibits a higher immunity to interference and interface structure sensitivity than the thrust and torque, and a higher recognition accuracy than the SED; (2) the adaptive matching between the driving parameters and drilling data leads to a significant 50.88% reduction in the error rate. In this paper, the adaptive indicator *SEL* is constructed based on the logistic function and the PSO algorithm, which is of significance for the accurate identification of the rock interface and the real-time sensing of roadway roof information.

**Figure 18:**
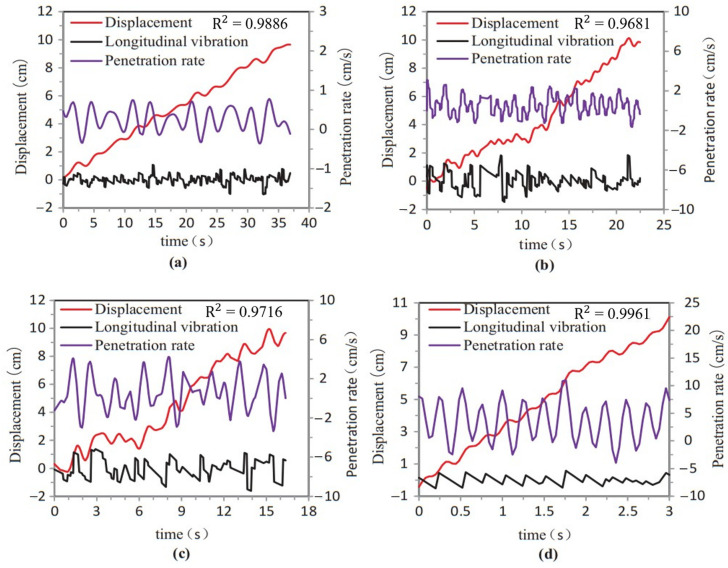
The displacement, longitudinal vibration, and drilling speed with different strengths, (**a**) rock strength: 82.58 MPa, (**b**) rock strength: 70.26 MPa, (**c**) rock strength: 68.69 MPa, (**d**) rock strength: 19.16 MPa.

Currently, rock structure detection primarily focuses on studying the thrust, torque, and drilling speed as direct indicators. However, these indicators are prone to significant fluctuations due to external factors [24]. Liu et al. [25] used the displacement, longitudinal vibration, and drilling speed as detection indicators. Figure 18 shows the time characteristic curves of the indicators during drilling. The data have been smoothed using a sinusoidal model in MATLAB; however, the overall volatility of the data remains relatively high. The curves do not exhibit clear interface response characteristics, and there is no apparent distinction between the interface stage and the non-interface stage. In this study, the characteristic indicator *SEL* is modulated based on the logistic function, as depicted in the C-SEL curve in Figure 14. While maintaining low volatility during the stable drilling phase, the *SEL* exhibits abrupt changes in the interface phase that significantly exceed the distribution levels observed in other phases. The heightened anti-interference properties and enhanced interface sensitivity of the *SEL* indicator facilitate the more effective detection and localization of interfaces.

Direct indicators such as the thrust and torque have advantages in data collection. But they are highly susceptible to ill-defined fluctuations due to drilling setup changes. For example, an increase in drilling speed will result in an increase in thrust and torque when drilling in the same strength of rock formation. However, there is no specific mathematical relationship between the trends of multiple indicators, causing an increase in the overall volatility of the data and affecting the identification accuracy. The *SEL* belongs to the specific energy class of indicators; the size of the data only exists in correlation with the nature of the rock formation itself and it does not appear to change with the change of drilling settings, in principle. Compared with direct indicators, the *SEL* avoids the data fluctuations caused by the change in drilling settings during the drilling process, and has stronger anti-interference and rock property correlation.

**Figure 19:**
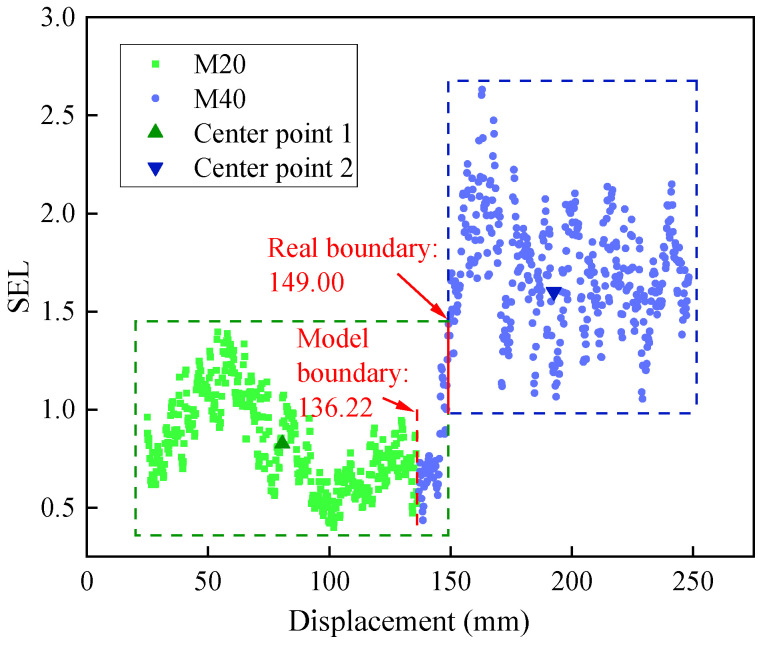
K-means clustering model recognition effect.

In the field of drill-following recognition, numerous scholars have utilized various intelligent algorithms for interface recognition and achieved notable results. For instance, Liu et al. [25] employed the K-means clustering algorithm to identify interfaces and observed a strong correlation between drilling speed and interface responsiveness. However, the majority of recognition models tend to only apply algorithms without further optimization based on specific research problem characteristics and test data properties. This study improves the fit of data to the CUSUM algorithm model by constructing a feature index and optimizing driving parameters to achieve a higher recognition accuracy. To demonstrate the effectiveness of our targeted optimization approach, the data from test group 1-450-1.0 are applied to the K-means clustering model, and Figure 19 shows the results.

The K-means clustering model shown in Figure 19 applies the algorithmic principle to divide the SEM data into drilling data at intensities of 20 MPa and 40 MPa. The boundary for the data corresponding to two strengths indicates the interface of the rock layer, which is observed at a displacement of 136.22 mm. It deviates by 12.78 mm from the actual interface position of 149.00 mm. This discrepancy can be attributed to how the K-means clustering algorithm divides centroids and classifies data based on the minimum distance within classes and the maximum distance between classes.

The high-intensity drilling data exhibit a higher level of discreteness compared to low-intensity data, resulting in more pronounced fluctuations within their distribution. Consequently, this leads to a larger intra-class distance range for high-intensity data than that found in low-intensity data. As a result, there is a forward shift in the detection position of interfaces. The K-means clustering algorithm utilizes data classification to search for intensity boundaries. However, this approach often results in the non-interface stage dominating the interface detection model. Consequently, it is susceptible to the issue where drilling states during the non-interface stage can influence the accurate detection of interface positions.

The CUSUM algorithm employed in this study is a change point detection model that specifically focuses on feature detection for the research problem. The modulation of the SED through the logistic function enhances the characterization of the interface and improves the fit with the CUSUM algorithm. Figure 16 displays the cumulative sum curves, where noticeable response fluctuations are observed near the interface position. In contrast, the curves for the other stages remain relatively stable, which minimizes interference with interface position detection. Furthermore, this study applies the PSO algorithm to continuously optimize the driving parameters based on data characteristics. This optimization process further enhances the interfacial sensitivity of indexes within the model. Figure 18 illustrates a comparison between the curves before and after optimization, demonstrating the positive impact of this optimization. The optimization for self-adaptive matching reduces interference from the non-interface stage and minimizes potential abnormal fluctuations to effectively improve the recognition accuracy.

However, this study also has certain research limitations. By reflecting the drilling characteristics through data intervals of a specific length, the model’s resistance to interference is enhanced. However, this approach may result in a reduction in the upper limit of accuracy for interface detection and localization compared to a directly analysis of the data. As a consequence, the CUSUM monitoring model, which is based on the interval coefficient of variation, can only identify the corresponding displacement of the interface interval. It does not have the capability to further localize the interface within the interval. The subsequent analysis will focus on examining the scale effect of drilling intervals based on the characteristics of drilling index data. By studying the anti-interference properties and interface sensitivity corresponding to different length intervals within the drilling data, we aim to ensure maximum accuracy and stability in our detection model.

## 7. Conclusions

(1) This study proposed the stratum interface detection method, which is based on the data characteristics observed during on-site drilling and the principles governing interface recognition. By constructing a CUSUM detection model to monitor characteristic mutation points in the interval coefficient of variation, this approach effectively located interfaces within rock strata.

(2) Based on the SED, the interface characterization indicator *SEL* was constructed through the logistic function. The findings indicated that the *SEL* exhibits superior anti-interference properties and interface structure sensitivity compared to thrust and torque measurements. Moreover, it demonstrates a higher accuracy in identifying interfaces than the SED. 

(3) Through an analysis of the mechanism of separating different drilling stages according to the driving parameters a and c, the recognition model was further optimized using the PSO algorithm. The results demonstrated a significant improvement in accuracy, with the average recognition error reduced from 2.83 mm to 1.39 mm, corresponding to a reduction in error rate of 50.88%. This improvement is attributed to the adaptive optimizing matching degree between driving parameters and data features using the PSO algorithm. 

(4) The detection of interfaces serves as a crucial prerequisite for the real-time sensing of geological changes in the roof. In this study, the adaptive indicator *SEL* is constructed based on the logistic function and the PSO algorithm, to achieve the precise detection of interfaces. This research holds significant importance in providing real-time insights into geological changes within coal mine rock, allowing for the timely optimization of support parameters and the proactive mitigation of roof collapse.

## Figures and Tables

**Table 1:** The strength conditions and interface positions of the samples.

Sample	Design UCS/MPa	Test UCS/MPa	Average Strength/MPa	Strength Difference/MPa	Interface Position/mm
1	20 × 40	11.85 × 37.46	24.66	25.61	149.00
2	30 × 50	28.41 × 49.38	38.90	20.97	156.60
3	30 × 40	28.41 × 37.46	32.94	9.05	150.50

**Table 2:** Comparison of different indicators for group 1-450-1.0.

Indicators	Mean Value of Interval Coefficient of Variation	Number of High Volatility Points	Detection Error/mm
SED	2.21 × 10^−2^	4	9.28
*SEL*	1.04 × 10^−2^	3	1.78

**Table 3:** The results of the interface identification.

Sample	UCS /MPa	Interface Position/mm	Drilling Speed/mm/s	*SEL*		SED	
				Detection/mm	Error/mm	Detection/mm	Error/mm
1	20 × 40	149.00	0.5	149.75	0.75	152.27	3.27
			0.8	149.75	0.75	144.71	4.29
			1	147.22	1.78	139.72	9.28
2	30 × 50	156.60	0.5	158.95	2.35	161.40	4.80
			0.8	153.88	2.72	163.99	7.39
			1	158.95	2.35	151.36	5.24
3	30 × 40	150.50	0.5	147.65	2.85	145.13	5.37
			0.8	157.69	7.19	157.69	7.19
			1	155.19	4.69	162.69	12.19
Average error/mm				2.83		6.56	

**Table 4:** Interface recognition errors before and after optimization.

Sample	Drilling Speed/mm/s	Detection Error/mm	
		Before Optimization	After Optimization
1	0.5	0.75	0.75
	0.8	0.75	0.75
	1	1.78	0.75
2	0.5	2.35	0.20
	0.8	2.72	2.35
	1	2.35	2.35
3	0.5	2.85	2.17
	0.8	7.19	2.85
	1	4.69	0.33
Average error/mm		2.83	1.39

## Data Availability

Some or all data, models, or code generated or used during the study are proprietary or confidential in nature and may only be provided with restrictions.
